# Sozial gerecht: Gesundheit – Umwelt – Klima: Tagungsbericht zum Kongress Armut und Gesundheit 2024

**DOI:** 10.1007/s00103-024-03936-z

**Published:** 2024-08-14

**Authors:** Marion Amler, Nicole Böhme, Marina Martin, Jens Hoebel

**Affiliations:** 1 Geschäftsstelle Berlin, Gesundheit Berlin-Brandenburg e. V., Berlin, Deutschland; 2 Fachgebiet Soziale Determinanten der Gesundheit, Robert Koch-Institut, Nordufer 20, 13353 Berlin, Deutschland

Was haben Armut, Gesundheit, Umwelt, Klima und soziale Gerechtigkeit miteinander zu tun? Und wie können wir unter Berücksichtigung aktueller Klimaveränderungen sozial gerechtere, gesundheitsförderliche Rahmenbedingungen herstellen? Um diese Fragen drehten sich die Diskussionen beim diesjährigen Kongress Armut und Gesundheit in Berlin-Dahlem, zu dem ca. 1900 Teilnehmende virtuell und in Präsenz an der Freien Universität Berlin zusammenkamen. An insgesamt 3 Tagen tauschten sich Vertreter*innen aus Wissenschaft, Praxis, Politik und Zivilgesellschaft in 115 Fachforen, Diskussionsrunden und Workshops aus, analog und digital.

Der Kongress war und ist eine Gemeinschaftsinitiative. Neu war in diesem Jahr – passend zum Schwerpunkt – die Kooperation mit dem Umweltbundesamt, welches neben der Deutschen Gesellschaft für Public Health (DGPH), der Berlin School of Public Health (BSPH) und der Freien Universität (FU) Berlin Mitveranstalter des Kongresses war. Ausgerichtet wird der Kongress seit 1995 von Gesundheit Berlin-Brandenburg e. V. Ohne die Unterstützung vielfältiger Kooperationspartner wäre der größte regelmäßig stattfindende Public-Health-Kongress Deutschlands nicht umzusetzen.^1^

Die einmalige Zusammensetzung seiner Teilnehmenden ist ein wesentliches Merkmal des Kongresses Armut und Gesundheit. Hier kommt die Mitarbeiterin einer Notunterkunft für Menschen mit Fluchterfahrungen zusammen mit dem Stadtplaner, einem Angestellten der regionalen Wohnungsbaugesellschaft, der Public-Health-Wissenschaftlerin, dem Studenten für soziale Arbeit und der Bezirkspolitikerin. Dabei nehmen die Themen und Perspektiven gleichberechtigt ihren Platz in den Diskussionen ein. Es entstehen gegenseitige Lernerfahrungen, Diskussionen auf Augenhöhe und Konzepte, die die Lebensrealitäten der Menschen aufgreifen und somit verändern können. So will der Kongress beitragen zum Austausch mit Betroffenen einerseits (hierfür ist das inzwischen fest etablierte „Gremium von Menschen mit Armutserfahrung“^2^ zentral) und zum Wissenschafts-Praxis-Transfer andererseits. Der Kongress versteht sich zudem als Nachwuchskongress und bietet eigens für Studierende entwickelte Formate wie den Science Slam an (vgl. hierzu das Selbstverständnis des Kongresses)^3^.

## Daten zum Zusammenhang zwischen Armut und Gesundheit

Armut stellt eine fundamentale Ursache von Krankheit und vorzeitiger Sterblichkeit dar. Seit Jahrzehnten zeigen Befunde u. a. des Robert Koch-Instituts (RKI) übereinstimmend, dass auch hierzulande Menschen in sozioökonomisch benachteiligten Verhältnissen besonders häufig von gesundheitlichen Beeinträchtigungen und schwerwiegenden chronischen Erkrankungen betroffen sind, was sich letztlich in einer früheren Sterblichkeit widerspiegelt [1–3]. Neue Befunde für Deutschland zeigen, dass sich diese gesundheitliche Ungleichheit in den letzten Jahrzehnten weiter vergrößert hat [3–5]. Gerade erst hat der Paritätische Gesamtverband seinen aktuellen Armutsbericht [6] veröffentlicht: Demnach lebten 2022 16,8 % der Bevölkerung Deutschlands in Armut. Am stärksten von Armut betroffen sind Alleinerziehende, kinderreiche Familien, Erwerbslose, Menschen mit einer niedrigen formalen Bildung und solche mit einem sogenannten Migrationshintergrund. Mit 21,8 % der Kinder und Jugendlichen war mehr als jedes 5. Kind von Armut betroffen. Die kurz darauf veröffentlichte Expertise zu den Erstergebnissen des Mikrozensus zur Armutsentwicklung 2023 [7] gab bezüglich des Rekordwertes der Kinderarmut im Vorjahr wieder Entwarnung, dennoch verharrt die Armut in Deutschland auf hohem Niveau und betrifft zunehmend auch Ältere und hier vor allem Frauen.

Aktuelle Daten zum Zusammenhang von Armut, sozialer Benachteiligung und Gesundheit wurden auf dem Kongress u. a. in der Reihe „Soziale Determinanten“ präsentiert. In einem Fachforum zeigten Forschende des INHECOV-Projekts („Socioeconomic inequalities in health during the COVID-19 pandemic“), einem Verbundprojekt von RKI, Universitätsklinikum Düsseldorf, Universität Köln und der Akademie für Öffentliches Gesundheitswesen, dass Geringqualifizierte und Beschäftigte in niedrigen beruflichen Positionen in der Pandemie besonders hohe Risiken für Infektionen und schwere COVID-19-Verläufe hatten [8–10]. Dazu kam, dass psychosoziale Belastungen durch die Pandemie in benachteiligten Gruppen besonders ausgeprägt waren [11]. Derzeit werden im Projekt Konzepte erarbeitet, um diese Ergebnisse in die Weiterbildung im öffentlichen Gesundheitsdienst einzubringen.

In einem zweiten Fachforum wurden gesundheitliche Ungleichheiten in der Pandemie bei Kindern, Jugendlichen und Familien beleuchtet. Daten des Bundesinstituts für Bevölkerungsforschung (BiB) verdeutlichten, dass ökonomische Unsicherheiten in der Pandemie mit einer geringeren Lebenszufriedenheit von Eltern einhergingen [12]. Noch nicht veröffentlichte RKI-Daten zeigten, dass Kinder und Jugendliche aus armutsgefährdeten Familien seltener an außerschulischen Sportangeboten teilnahmen, während dies für die Teilnahme an Sport-AGs im schulischen Kontext nicht galt.

Das dritte Fachforum beschäftigte sich mit Gesundheitsfolgen von Rassismus und Diskriminierung, einem in Deutschland noch jungen Forschungsfeld. Anhand von Praxisberichten der Antidiskriminierungsstelle des Bundes und Studiendaten wurden Diskriminierungsrisiken im Gesundheitswesen beschrieben. Eine Studie, an der u. a. das Deutsche Zentrum für Integrations- und Migrationsforschung beteiligt war, zeigte, dass Personen mit in Deutschland verbreiteten Namen gegenüber Personen mit in der Türkei oder Nigeria verbreiteten Namen bei der Terminvergabe von Praxen bevorteilt werden [13]. Eine RKI-Studie ergab, dass Diskriminierungserfahrungen im Alltag mit erhöhten Herz-Kreislauf-Risiken einhergehen und dies bei einem geringeren Zugehörigkeitsgefühl zur Gesellschaft in Deutschland verstärkt wird (noch nicht veröffentlicht).

Die AG Sozialepidemiologie, eine gemeinsame Arbeitsgruppe der Deutschen Gesellschaft für Medizinische Soziologie e. V. (DGMS), der Deutschen Gesellschaft für Sozialmedizin und Prävention e. V. (DGSMP) und der Deutschen Gesellschaft für Epidemiologie e. V. (DGEpi), veranstaltete ein Fachforum zusammen mit der AG Health Geography und der AG Krebsepidemiologie der DGEpi. Hier wurden Konzepte und Daten zu sozialräumlichen Ansatzpunkten für die Prävention diskutiert. Deutlich wurde, dass Merkmale des Sozialraums, z. B. der Region oder Nachbarschaft, eigenständige Bedeutungen für die Krankheitsrisken der Menschen haben können, die über Gesundheitsfolgen individueller Armutslagen hinausgehen. Die Vernetzung lokaler Akteur*innen und der Austausch zwischen Wissenschaft, Praxis, Politik und den Menschen vor Ort wurden für wichtig erachtet, um lokal spezifische Präventionspotenziale zu identifizieren.

## Vorstellung der HBSC-Studie auf der Satellitentagung

Neben aktuellen Daten des Robert Koch-Institutes wurden auf der Satellitentagung am 04.03.2024 Ergebnisse der internationalen HBSC-Studie (Health Behaviour in School-aged Children) für Deutschland vorgestellt.^4^ Die Studie erfasst alle 4 Jahre die Gesundheit von Schüler*innen und nimmt dabei Aspekte wie körperliche Aktivität, Mobbing und Cybermobbing, psychisches Wohlbefinden, Gesundheitskompetenz und gesundheitliche Ungleichheiten in den Blick [14].

Aus den formulierten Bedarfen wurden notwendige Maßnahmen abgeleitet. Das gesunde Aufwachsen von Kindern und Jugendlichen im Schulalter wurde dabei als gesamtgesellschaftliche Aufgabe definiert. Zudem wurden Vorgehensweisen und „Leuchtturmprojekte“ im Sinne des Ansatzes „Health in All Policies“ vorgestellt und diskutiert. Dabei ging es nicht nur darum, die Folgen von Armut zu mildern, sondern auch die Ursachen anzugehen und dafür Impulse in Politikbereiche außerhalb des Gesundheitsressorts zu geben.

Eine intensive Pressearbeit im Vorfeld der Satellitentagung weckte ein hohes Medieninteresse an den HBSC-Ergebnissen. Direkt nach der Pressekonferenz wurde das Fachpublikum informiert und im *Journal of Health Monitoring* wurden die Ergebnisse am selben Tag veröffentlicht. Die große Resonanz hat gezeigt, dass die Verbreitung von Forschungsergebnissen immer mitgedacht werden sollte.

## Der Klimawandel als neue Facette der sozialen Frage

Das Thema Health in All Policies zog sich wie ein roter Faden durch das Kongressprogramm, ebenso wie die Notwendigkeit gesellschaftlicher Transformationsprozesse beispielsweise im Zusammenhang mit dem Klimawandel. Damit knüpften die Diskussionen unmittelbar an die Themen des Kongresses 2023 an [15].

Bundesgesundheitsminister Prof. Dr. Karl Lauterbach bezeichnete den Klimawandel in seiner Eröffnungsrede^5^ als eine neue Facette der sozialen Frage: „Der Klimawandel ist ein Faktor, der die Nachteile, die arme Menschen haben …, in allen Bereichen nur verstärken wird. Das, was an Armut jetzt schon einschränkend auf die Lebensqualität und Lebenserwartung wirkt, wird umso stärker wirken durch den Klimawandel. Das ist eine neue soziale Frage.“ Eine, die sich nicht getrennt von den zunehmenden gesellschaftlichen Ungleichheiten betrachten ließe: „Es sind diejenigen, die ohnedies durch Vermögen und Einkommen schon privilegiert sind, die einen großen Anteil am Klimawandel haben, unter denen der größte Teil der ärmeren Bevölkerung besonders leidet.“ Als mögliche Lösungen nannte der Minister u. a. die Einrichtung von Gesundheitskiosken in den ärmsten 1000 Stadtteilen Deutschlands als niedrigschwellige Anlaufpunkte und die Bürgerversicherung für alle – denn diese „unerträgliche Ungerechtigkeit“ sei ein „Makel unseres Gesundheitssystems“. Vorhaben dieser Art stoßen jedoch, so Lauterbach, im Politikbetrieb oft auf Ressentiments und seien in der derzeitigen Koalition nicht durchsetzbar.

Prof. Dr. Alena Buyx, Vorsitzende des Deutschen Ethikrates, stellte das Konzept der Vulnerabilität [16] in den Fokus ihrer Keynote. Sie evozierte dabei das Bild eines Dreiecks aus sozialer Lage, Gesundheit und Klima, in dessen Zentrum eine steigende Vulnerabilität stehe: „Wer arm ist, ist auch klimakränker“ – dies sei eine enorme Herausforderung. Als Gesamtstrategie dagegen brauche es positive Zukunftsbilder: „Wir brauchen die guten Geschichten!“ Dafür böte es sich an, die Themen Gesundheit und Klima miteinander zu vereinen. Denn, so Buyx im anschließenden Gespräch mit Prof. Dr. Maja Göpel: „Dinge, die fürs Klima gut sind, sind oft auch für die Gesundheit gut.“ Unmittelbar im Nachgang des Kongresses wurde am 13.03. die Stellungnahme „Klimagerechtigkeit“ vom Deutschen Ethikrat veröffentlicht [17].

## Wie wird medial über das Thema Armut und Gesundheit berichtet?

Eine nachhaltige Verankerung des Themas Armut im öffentlichen Diskurs von Public Health sowie die Besetzung des Themas Gesundheit im Diskurs zu Armut ist ein zentrales Anliegen des Kongresses. Ein Panel setzte sich in diesem Jahr deshalb mit der Medienberichterstattung zum Thema Armut und Gesundheit auseinander. Im Rahmen einer Podiumsdiskussion wurde diskutiert, wie über den Themenkomplex auf angemessene Weise berichtet werden kann. Einigkeit bestand darin, dass der Ton in den sozialen Medien immer rauer werde. Mehrere armutsbetroffene Teilnehmende berichteten von ihren negativen Erfahrungen auf unterschiedlichen Plattformen, die von Schuldzuweisungen bis hin zu Beschimpfungen reichten. Problematisiert wurden zudem Begrifflichkeiten wie „sozial schwach“ und „bildungsfern“. Besser sei es, von finanziell benachteiligten oder chancenbeschnittenen Personen zu sprechen. In der Berichterstattung müsse die Darstellung persönlicher Schicksale immer gerahmt werden durch strukturelle Ursachen von Armut und Krankheit. Die Wissenschaft sei in der Pflicht, bei der Erfassung und Veröffentlichung ihrer Daten zu prüfen, wie sich komplexe Inhalte vereinfachen und strukturelle Ursachen von Armut und Krankheit konkret benennen ließen.^6^

## Menschen ohne Krankenversicherung

Zur gesundheitlichen Lage und Versorgung von Menschen ohne Krankenversicherung gab es mehrere Veranstaltungen, in denen Betroffene, Wissenschaftler*innen, Praktiker*innen und Personen aus der Verwaltung in den Austausch kamen. Trotz bestehender Krankenversicherungspflicht seien nach Schätzungen Hunderttausende Menschen ohne Krankenversicherung, so Janina Gach (Ärzte der Welt). Die Zahl wurde mehrfach kontrovers diskutiert. Kritisch wurde ins Feld geführt, dass es auch Schätzungen gäbe, die bei über einer Millionen Betroffenen liegen. Die Herausforderung, hier valide Zahlen anzuführen, bestehe darin, dass viele Betroffene nicht vom System erfasst würden. Sie wären oftmals wohnungslos, ohne Papiere, EU-Ausländer*innen oder auch Menschen mit Fluchthintergrund sowie illegalisierte Menschen. Letztere stünden vor besonderen Herausforderungen. Denn zwar gebe es laut Gesetzeslage einen Anspruch auf medizinische Versorgung auch für Menschen ohne legalen Aufenthaltsstatus, aber bei der Beantragung der Kostenübernahme zur medizinischen Behandlung unterlägen die zuständigen Ämter der gesetzlichen Übermittlungspflicht. Somit stünden diese Menschen vor dem Dilemma, sich entweder nicht behandeln zu lassen oder einer drohenden Abschiebung auszusetzen. In einem medizinischen Notfall müsste zwar eine Notversorgung im Krankenhaus unter Einhaltung der ärztlichen Schweigepflicht erfolgen, doch aufgrund von hohen rechtlichen Hürden bei der Refinanzierung dieser Leistung erfolge oftmals nur eine unzureichende Minimalversorgung. Auch Menschen ohne dieses Risiko wären stark unterversorgt, etwa Personen mit Beitragsschulden, die oftmals ehemals selbstständig oder privat versichert waren. Ohne gültige Versicherung würden sie in Praxen oder Kliniken teilweise abgewiesen oder zu früh entlassen. Gesetzliche Ausschlüsse (z. B. für EU-Bürger*innen), eine komplexe und unübersichtliche Rechtslage (gesetzliche und private Krankenversicherung, Sondersysteme) sowie Schnittstellenproblematiken führten dazu, dass viele Menschen ohne ausreichenden Versicherungsschutz in Deutschland leben und diese oft viel zu spät medizinisch notwendige Hilfe in Anspruch nehmen oder gar eine Ablehnung ihrer Behandlung erfahren. Dadurch käme es oftmals zu Chronifizierungen und schweren Krankheitsverläufen, die bei einer rechtzeigen Behandlung vermeidbar wären, ergänzte Louise Zwirner (Clearingstelle Berliner Stadtmission). Unterstützung werde daher oft ehrenamtlich geleistet und über Spenden finanziert, mitunter würden auch Kommunen und Länder entsprechende Projekte finanzieren.

Einige dieser Akteur*innen haben sich im Jahr 2022 zum Bundesverband Anonymer Behandlungsschein und Clearingstellen für Menschen ohne Krankenversicherung e. V. (BACK) zusammengeschlossen. Dieser setzt sich einerseits in der konkreten Versorgung, andererseits für eine politische Verbesserung der Rahmenbedingungen ein. Oberstes Ziel sei, dass kein Mensch mehr vom Zugang zu gesundheitlicher Versorgung ausgeschlossen wird, so Nele Wilk (Armut und Gesundheit e. V.). Aktuell erfolge die gesundheitliche Versorgung von nichtversicherten Menschen oft in unzureichenden und teils ehrenamtlich organisierten Parallelstrukturen oder durch Clearingstellen, welche die Rückkehr in die Regelversorgung unterstützen oder medizinische Behandlungen finanzieren. Die Vermittlungsquote in eine reguläre Absicherung liege bei etwa 31 %, so Noah Peitzmann (Anonymer Krankenschein Bonn e. V.), was sich durch die hohe Komplexität des Gesundheitssystems sowie durch teils unüberwindbare rechtliche Hürden erkläre. Der BACK fordere deshalb u. a. die Abschaffung der Trennung zwischen gesetzlicher und privater Krankenversicherung, die Abschaffung der Übermittlungspflicht nach § 87 Aufenthaltsgesetz und die Gleichstellung von EU-Bürger*innen.^7^ Bis die entsprechenden rechtlichen Grundlagen für einen diskriminierungsfreien Zugang zur Gesundheitsversorgung für alle geschaffen wurden, fordert der BACK als Übergangslösung die bundesweite Einrichtung von Clearingstellen mit der Möglichkeit, Kosten für medizinische Behandlungen zu übernehmen.

## Umweltgerechtigkeit

In 2 Veranstaltungen fokussierte die inter- und transdisziplinäre Arbeitsgruppe Gesundheitsfördernde Gemeinde- und Stadtentwicklung (AGGSE^8^) des Deutschen Instituts für Urbanistik gGmbH das Konzept der Umweltgerechtigkeit, welches bereits seit 20 Jahren diskutiert, aber noch kaum angewendet wird. Insbesondere in den USA sei das Thema deutlich weiter fortgeschritten, da es dort durch Bürgerrechtsbewegungen verstärkt auf die politische Agenda gebracht wurde, so Prof. Dr. Claudia Hornberg (Universität Bielefeld, Sachverständigenrat für Umweltfragen). Das Konzept nimmt die sozialräumlich ungleiche Verteilung von gesundheitsrelevanten Umweltbelastungen und -ressourcen unter Gerechtigkeitsaspekten in den Blick [18]. Hornberg führte aus, dass „Stadtteile mit niedrigerem sozioökonomischem Status im Durchschnitt 52 % weniger Grünflächen zur Verfügung haben als Stadtteile mit hohem sozioökonomischem Status“. Zudem zeigten auch internationale Metaanalysen, dass Menschen mit niedrigerem sozioökonomischen Status ein höheres hitzebedingtes Sterberisiko aufweisen als Personen mit höherem sozioökonomischen Status.

Die Städte Kassel und Marburg gaben Einblick in Umsetzung und Herausforderungen in der Praxis. Kassel ist eine der wenigen deutschen Städte, die – resultierend aus einer Koalition zweier Dezernate – bereits einen entsprechenden politischen Beschluss dazu haben. Dr. Anja Starick, Leiterin des Umwelt- und Gartenamtes der Stadt Kassel, berichtete, dass das Konzept die Möglichkeit biete, Handlungsbedarfe zu identifizieren sowie Umweltplanung und die sozialökologische Transformation voranzubringen. Allerdings gäbe es auch Hürden, etwa wenn straßenbauliche Maßnahmen in autofreundlichen Städten wie Kassel umgesetzt werden sollten. Peter Schmittdiel (Universitätsstadt Marburg) bestätigte diese Herausforderungen in der Anpassung und zeigte für Marburg auf, dass teilweise besonders belastete Gebiete nicht primär Hitze, sondern – bedingt durch deren Tallage – eher starker Beschattung und der Gefahr von Starkregenereignissen ausgesetzt seien.

## Öffentlicher Raum als Ressource für Gesundheitsförderung

Die Herausforderungen für Umwelt und Lebensqualität der Menschen, die mit der Verdichtung der Städte einhergehen, wurden in Veranstaltungen des Umweltbundesamtes diskutiert und ihr Potenzial als Ressource der Gesundheitsförderung herausgearbeitet. Mit dem Forschungsprojekt „Neues Europäisches Bauhaus weiterdenken: Nachhaltige Mobilität und resiliente Räume für mehr Lebensqualität“^9^ wird am Umweltbundesamt ein systematisches Review für die wissenschaftliche Evidenz von sozialer Ungleichheit bei der Exposition von umweltbezogenen Gesundheitsrisiken und dem Zugang zu Gesundheitsressourcen erarbeitet und damit eine Forschungslücke geschlossen, so Jascha Wiehn vom Umweltbundesamt. Ein Fokus liege dabei auf Kindern und Jugendlichen, die – bezogen auf ihr Körpergewicht – mehr Schadstoffe über die Atmung aufnehmen als Erwachsene, so Miriam Dross vom Fachbereich für nachhaltige Mobilität am Umweltbundesamt. Auf Grundlage der Studie zur Gesundheit von Kindern und Jugendlichen in Deutschland (KiGGS) und der Deutschen Umweltstudie zur Gesundheit (GerES) konnte in einem ersten Ergebnis das Konzept der Umweltgerechtigkeit bestätigt werden, denn für 36 % der Kinder mit niedrigem sozioökonomischen Status sei die Gesundheitsressource Grünfläche nicht auf kurzem Wege erreichbar.

Urbaner Raum sei ein knappes Gut und könne bei sinnvoller Neuverteilung zur Gesundheitsförderung beitragen. Derzeit dominiere der motorisierte Individualverkehr den öffentlichen Raum in deutschen Städten. Eine Schwerpunktverlagerung auf Fuß‑, Rad- und öffentlichen Verkehr käme nicht nur der Umwelt zugute, sondern erhöhe zugleich die Lebensqualität der Menschen. Krankheitskosten könnten gesenkt und Sozialversicherungssysteme entlastet werden, so Dross. In London etwa seien seit der Einführung einer Maut für die Innenstadt ca. 80.000 Autos weniger täglich unterwegs und die Anzahl der Radfahrer*innen sei um 2 Drittel gestiegen.^10^ Menschen fühlten sich sicherer, wodurch die aktive Alltagsmobilität als Gesundheitsressource gefördert würde. Momentan sei die Umverteilung des öffentlichen Raumes zwar rechtlich schwer umzusetzen; Anlass zur Hoffnung böte jedoch die geplante Novellierung der Straßenverkehrsordnung.

## Klima- und Umweltanpassungsmaßnahmen in der Praxis

Anhand des Berliner Luftreinhalteplans legte Andreas Kerschbaumer (Senatsverwaltung für Mobilität, Verkehr, Klimaschutz und Umwelt von Berlin) dar, wie mithilfe politischer Entscheidungen die Neuverteilung des öffentlichen Raumes als gesundheitsförderliche Ressource gestärkt werden kann. Derzeit würden die gesetzlich vorgeschriebenen Werte von 40 µg/m^3^ für Stickstoffdioxid im Jahresmittel eingehalten, die von der Weltgesundheitsorganisation (WHO) empfohlenen 10 µg/m^3^ allerdings nicht erreicht. Daher setze man auf 3 Bausteine: Durch den Ausbau von Radstreifen sei die Stickstoffbelastung um 22 % für Radfahrende zurückgegangen. Die Elektrifizierung der Busflotte (227 Elektrobusse Mitte 2023) führte zu einer Verringerung von 62 µg/m^3^ auf 19 µg/m^3^ im Jahresmittel am Berliner Hardenbergplatz und mit Tempo 30 verringerten sich Stickstoff- und Feinstaubbelastung um ca. 2 µg/m^3^.

Hitzeaktionspläne werden als umwelt- und gesundheitspolitische Maßnahme in den Kommunen und Regionen zukünftig etabliert und derzeit bereits in 5 Bundesländern und 8 deutschen Städten konkret erarbeitet, so Dr. Wolfgang Straff vom Umweltbundesamt. Der Hitzeaktionsplan der Stadt Dresden solle bereits bis 2025 vollständig angewandt werden. Marit Gronwald vom Gesundheitsamt Dresden konnte darlegen, dass neben verhältnispräventiven Maßnahmen (Aufstellen von Trinkbrunnen, bauliche Maßnahmen zur Reduzierung von Hitze in Innenräumen) vor allem die Kommunikation ein Kernelement für die Umsetzung darstellt. So werden z. B. in Kooperation mit dem öffentlichen Verkehrsnetz an heißen Tagen Hitzetipps im Fahrgastfernsehen veröffentlicht, ein Kinderheft zum Thema Hitze ist in Vorbereitung und eine Anlaufstelle für Hitzeschutz wird eingerichtet. Bereits im letzten Jahr wurde das Hitze-Handbuch erstellt, auf dem in diesem Jahr Schulungen mit einer lokalen Bildungsakademie aufbauen. Wie die partizipative Planung für einen gesundheitsförderlichen öffentlichen Raum aussehen kann, konnte Pat Bohland (LIFE Bildung Umwelt Chancengleichheit e. V.) zeigen. In Berlin-Neukölln wurden gemeinsam mit Bürger*innen 2 Gesundheitskarten erarbeitet.^11^ Darin wurden zum einen Mängel identifiziert, die es Menschen erschweren, zu Fuß in den Quartieren unterwegs zu sein. Zum anderen wurden Ideen zusammengetragen, wie Straßen für Fußgänger*innen sicherer, verkehrsberuhigter und barriereärmer werden können. Aus beiden Gesundheitskarten wurden Handlungsempfehlungen für ein kühles und barrierefreies Wegenetz erarbeitet zur Verwendung für Entscheidungsträger*innen in Politik, Verkehrs‑, Umwelt- und Stadtplanung.

## Neue Allianzen am Beispiel des Themas „innere Sicherheit“

In der Veranstaltung „We can work it out: Interdisziplinäre Perspektiven für gesellschaftliche Herausforderungen“^12^ wurde anhand der Themen Klima, Gesundheit und Sicherheit diskutiert, wo intersektorale Vernetzung bereits gut gelingt, wo nicht und wie diese ausgebaut werden könnte. Erich Marks (Deutscher Präventionstag) unterschied explizite und implizite Ansätze der Kriminalprävention: „Was wir explizit tun können, ist, Fahrräder, Autos und Türen abschließen etc. Wer aber gute Kriminalprävention machen will, der muss sich um die Armut in der Gesellschaft kümmern, um soziale Verhältnisse, Bildung, Frühe Hilfen … Vermutlich 80 % des Potenzials liegen im Impliziten“. Lea Dohm (Deutsche Allianz Klimawandel und Gesundheit) machte deutlich, dass die Klimakrise eine Gesundheitskrise sei und dass der Klimawandel ein Sicherheitsrisiko darstelle, da Verteilungsfragen noch stärker hervortreten und Hitze auch die Aggressivität steigere. Stefan Bräunling (Kooperationsverbund Gesundheitliche Chancengleichheit) betonte: „Gesundheitsförderung ist Demokratieförderung. Prävention ist Demokratieförderung. Und Klimaschutz ist Demokratieförderung“.

## Ökosalute Referate in allen Ministerien

Auch in der Abschlussveranstaltung^13^ wurde Umweltschutz als Voraussetzung für Gesundheit und Freiheit sowie als Demokratieschutz diskutiert. Prof. Dr. Dirk Messner, Präsident des Umweltbundesamtes, fasste zusammen, was im Laufe der 3 Kongresstage immer wieder deutlich geworden war: dass gerade im lebensweltlichen Bereich die Co-Benefit-Potenziale zwischen Umwelt, Gesundheit und Lebensqualität besonders groß seien, zugleich aber auch die Polarisierungspotenziale. Er stellte fest: „Im Augenblick gewinnen wir in ganz Europa keine demokratischen Wahlen mit dem Projekt der sozialökologischen Transformation. … Wir sind in unserer Kommunikation nicht gut genug. Das ist der Tatbestand.“

Prof. Dr. Raimund Geene adressierte gesellschaftliche Ungerechtigkeiten: „Was wir nicht machen, ist, radikal die Eigentumsverhältnisse und die Ungerechtigkeiten zu thematisieren.“ In einem eigenen Panel hatten Vertreter*innen von mein Grundeinkommen e. V., taxmenow – Initiative für Steuergerechtigkeit e. V., dem Deutschen Institut für Wirtschaftsforschung und dem Deutschen Gewerkschaftsbund über genau diese Ungerechtigkeiten diskutiert und sich damit weg von der für Public Health typischen Perspektive der Armutsfolgenprävention hin zur Bekämpfung ihrer Ursachen bewegt, wenn auch mit unterschiedlichen Lösungsansätzen.^14^

Dr. Kirsten Kappert-Gonther kritisierte, dass die Verantwortung für Transformationen allzu oft auf das Individuum abgewälzt werde: „Wenn wir über Gerechtigkeit sprechen, neigen wir dazu, die individuelle Verantwortung für gesundheitsfreundliches Verhalten stark zu machen. Wir laden das häufig beim Individuum ab, wo es zuvörderst nicht hingehört. Es gehört in die Verhältnisse. Und das sind politische Aufgaben: die Lebenswelten so zu gestalten, dass möglichst viele Menschen sich gut und sicher und gesund entwickeln können.“

Auf die Frage, wie sich dies politisch umsetzen ließe, sprach sich Messner für nichts Geringeres als eine Reorganisation der Politik aus: „Wenn sich die Welt verändert, dann muss sich auch die Organisation der Politik verändern.“ Er plädierte für ein integratives Zusammendenken von gesundheitlichen, ökologischen und Well-being-Dimensionen. Das verlange mindestens eine stärkere Koordination aller Ressorts und bestenfalls die Einrichtung ökosaluter Referate in allen Ministerien.

## Ausblick 2025

Im kommenden Jahr begeht der Kongress sein 30-jähriges Jubiläum. In dieser Hinsicht wird es für die Organisator*innen ein besonderes Jahr. Das Thema der sozialökologischen Transformation wird uns weiter begleiten. Fest stehen außerdem bereits Zeitpunkt und Ort: Der 30. Kongress Armut und Gesundheit wird am 17. und 18.03.2025 im Henry-Ford-Bau der Freien Universität in Berlin-Dahlem stattfinden. Wir laden Sie schon jetzt herzlich ein, daran teilzunehmen!

## Footnotes

1 Förderung des Kongresses: https://www.armut-und-gesundheit.de/partner (Zugriff: 24.05.2024).

2 https://www.armut-und-gesundheit.de/kongress-2024/gremium-menschen-mit-armutserfahrungen (Zugriff: 04.06.2024).

3 https://www.armut-und-gesundheit.de/ueber-den-kongress/selbstverstaendnis (Zugriff: 24.05.2024).

4 https://www.gesundheitliche-chancengleichheit.de/satellit-armut-gesundheit/satellit-2024/ (Zugriff: 10.07.2024).

5 Eröffnungsveranstaltung: https://www.youtube.com/watch?v=x3vxbszBxB8  (Zugriff 24.05.2024).

6 Ergebnisse des Panels: https://www.armut-und-gesundheit.de/uploads/tx_gbbkongressarchiv/177_Amler_PP.pdf (Zugriff 24.06.2024).

7 https://anonymer-behandlungsschein.de/gemeinsame-politische-ziele-des-back/ (Zugriff: 03.06.2024).

8 https://difu.de/arbeitsgruppe-gesundheitsfoerdernde-gemeinde-und-stadtentwicklung (Zugriff: 03.06.2024).

9 www.umweltbundesamt.de/forschungsprojekt-adneb-neues-europaeisches-bauhaus (Zugriff 09.06.2024).

10 https://www.zeit.de/mobilitaet/2019-05/citymaut-strassenverkehr-co2-reduzierung-london-kamerasysteme-datenschutz (Zugegriffen: 08.07.2024).

11 Vgl. unter https://life-online.de/project/gesundheitskarte-neukoelln/ (Zugriff: 10.07.2024).

12 https://www.armut-und-gesundheit.de/uploads/tx_gbbkongressarchiv/2024_We_can_work_it_out.pdf (Zugriff: 05.08.2024).

13 Abschlussveranstaltung: https://www.youtube.com/live/SF3LVArlCDU (Zugriff: 08.07.2024).

14 https://www.armut-und-gesundheit.de/uploads/tx_gbbkongressarchiv/2024_Ungleichheit_bekaempfen.pdf (Zugriff: 05.08.2024).
